# Supplementary material for: Genome-wide analysis of MADS-box transcription factor gene family in wild emmer wheat (Triticum turgidum subsp. dicoccoides)
Source: PLoS One. 2024 Mar 7;19(3):e0300159. doi: 10.1371/journal.pone.0300159 (PMC10919676; doi:10.1371/journal.pone.0300159)
Supplement: S1 Fig — Conserved domain predictions for the 117 T. dicoccoides MADS-box proteins are presented. (PDF) [file pone.0300159.s001.pdf]

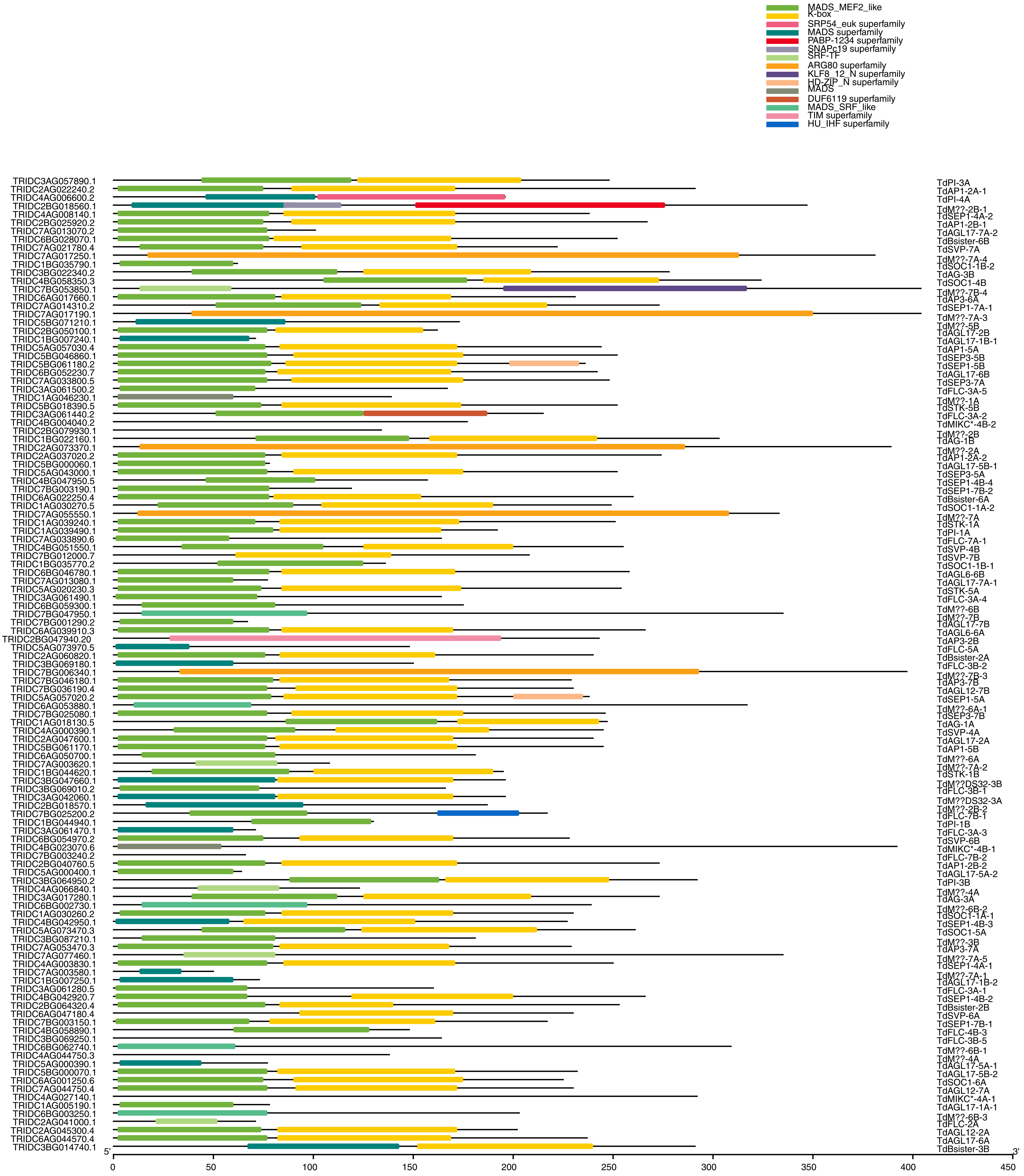
MADS_MEF2_like
K-box
SRP54_euk superfamily
MADS superfamily
PABP-1234 superfamily
SNAPc19 superfamily
SRF-TF
ARG80 superfamily
KLF8_12_N superfamily
HD-ZIP_N superfamily
MADS
DUF6119 superfamily
MADS_SRF_like
TIM superfamily
HU_IHF superfamily
TRIDC3AG057890.1
TRIDC2AG022240.2
TRIDC4AG006600.2
TRIDC2BG018560.1
TRIDC4AG008140.1
TRIDC2BG025920.2
TRIDC7AG013070.2
TRIDC6BG028070.1
TRIDC7AG021780.4
TRIDC7AG017250.1
TRIDC1BG035790.1
TRIDC3BG022340.2
TRIDC4BG058350.3
TRIDC7BG053850.1
TRIDC6AG017660.1
TRIDC7AG014310.2
TRIDC7AG017190.1
TRIDC5BG071210.1
TRIDC2BG050100.1
TRIDC1BG007240.1
TRIDC5AG057030.4
TRIDC5BG046860.1
TRIDC5BG061180.2
TRIDC6BG052230.7
TRIDC7AG033800.5
TRIDC3AG061500.2
TRIDC1AG046230.1
TRIDC5BG018390.5
TRIDC3AG061440.2
TRIDC4BG004040.2
TRIDC2BG079930.1
TRIDC1BG022160.1
TRIDC2AG073370.1
TRIDC2AG037020.2
TRIDC5BG000060.1
TRIDC5AG043000.1
TRIDC4BG047950.5
TRIDC7BG003190.1
TRIDC6AG022250.4
TRIDC1AG030270.5
TRIDC7AG055550.1
TRIDC1AG039240.1
TRIDC1AG039490.1
TRIDC7AG033890.6
TRIDC4BG051550.1
TRIDC7BG012000.7
TRIDC1BG035770.2
TRIDC6BG046780.1
TRIDC7AG013080.1
TRIDC5AG020230.3
TRIDC3AG061490.1
TRIDC6BG059300.1
TRIDC7BG047950.1
TRIDC7BG001290.2
TRIDC6AG039910.3
TRIDC2BG047940.20
TRIDC5AG073970.5
TRIDC2AG060820.1
TRIDC3BG069180.1
TRIDC7BG006340.1
TRIDC7BG046180.1
TRIDC7BG036190.4
TRIDC5AG057020.2
TRIDC6AG053880.1
TRIDC7BG025080.1
TRIDC1AG018130.5
TRIDC4AG000390.1
TRIDC2AG047600.1
TRIDC5BG061170.1
TRIDC6AG050700.1
TRIDC7AG003620.1
TRIDC1BG044620.1
TRIDC3BG047660.1
TRIDC3BG069010.2
TRIDC3AG042060.1
TRIDC2BG018570.1
TRIDC7BG025200.2
TRIDC1BG044940.1
TRIDC3AG061470.1
TRIDC6BG054970.2
TRIDC4BG023070.6
TRIDC7BG003240.2
TRIDC2BG040760.5
TRIDC5AG000400.1
TRIDC3BG064950.2
TRIDC4AG066840.1
TRIDC3AG017280.1
TRIDC6BG002730.1
TRIDC1AG030260.2
TRIDC4BG042950.1
TRIDC5AG073470.3
TRIDC3BG087210.1
TRIDC7AG053470.3
TRIDC7AG077460.1
TRIDC4AG003830.1
TRIDC7AG003580.1
TRIDC1BG007250.1
TRIDC3AG061280.5
TRIDC4BG042920.7
TRIDC2BG064320.4
TRIDC6AG047180.4
TRIDC7BG003150.1
TRIDC4BG058890.1
TRIDC3BG069250.1
TRIDC6BG062740.1
TRIDC4AG044750.3
TRIDC5AG000390.1
TRIDC5BG000070.1
TRIDC6AG001250.6
TRIDC7AG044750.4
TRIDC4AG027140.1
TRIDC1AG005190.1
TRIDC6BG003250.1
TRIDC2AG041000.1
TRIDC2AG045300.4
TRIDC6AG044570.4
TRIDC3BG014740.1
TdPI-3A
TdAP1-2A-1
TdPI-4A
TdM??-2B-1
TdSEP1-4A-2
TdAP1-2B-1
TdAGL17-7A-2
TdBsister-6B
TdSVP-7A
TdM??-7A-4
TdSOC1-1B-2
TdAG-3B
TdSOC1-4B
TdM??-7B-4
TdAP3-6A
TdSEP1-7A-1
TdM??-7A-3
TdM??-5B
TdAGL17-2B
TdAGL17-1B-1
TdAP1-5A
TdSEP3-5B
TdSEP1-5B
TdAGL17-6B
TdSEP3-7A
TdFLC-3A-5
TdM??-1A
TdSTK-5B
TdFLC-3A-2
TdMIKC*-4B-2
TdM??-2B
TdAG-1B
TdM??-2A
TdAP1-2A-2
TdAGL17-5B-1
TdSEP3-5A
TdSEP1-4B-4
TdSEP1-7B-2
TdBsister-6A
TdSOC1-1A-2
TdM??-7A
TdSTK-1A
TdPI-1A
TdFLC-7A-1
TdSVP-4B
TdSVP-7B
TdSOC1-1B-1
TdAGL6-6B
TdAGL17-7A-1
TdSTK-5A
TdFLC-3A-4
TdM??-6B
TdM??-7B
TdAGL17-7B
TdAGL6-6A
TdAP3-2B
TdFLC-5A
TdBsister-2A
TdFLC-3B-2
TdM??-7B-3
TdAP3-7B
TdAGL12-7B
TdSEP1-5A
TdM??-6A-1
TdSEP3-7B
TdAG-1A
TdSVP-4A
TdAGL17-2A
TdAP1-5B
TdM??-6A
TdM??-7A-2
TdSTK-1B
TdM??DS32-3B
TdFLC-3B-1
TdM??DS32-3A
TdM??-2B-2
TdFLC-7B-1
TdPI-1B
TdFLC-3A-3
TdSVP-6B
TdMIKC*-4B-1
TdFLC-7B-2
TdAP1-2B-2
TdAGL17-5A-2
TdPI-3B
TdM??-4A
TdAG-3A
TdM??-6B-2
TdSOC1-1A-1
TdSEP1-4B-3
TdSOC1-5A
TdM??-3B
TdAP3-7A
TdM??-7A-5
TdSEP1-4A-1
TdM??-7A-1
TdAGL17-1B-2
TdFLC-3A-1
TdSEP1-4B-2
TdBsister-2B
TdSVP-6A
TdSEP1-7B-1
TdFLC-4B-3
TdFLC-3B-5
TdM??-6B-1
TdM??-4A
TdAGL17-5A-1
TdAGL17-5B-2
TdSOC1-6A
TdAGL12-7A
TdMIKC*-4A-1
TdAGL17-1A-1
TdM??-6B-3
TdFLC-2A
TdAGL12-2A
TdAGL17-6A
TdBsister-3B
5'
3'
0
50
100
150
200
250
300
350
400
450
