# Supplementary figures and images for: Genome-wide analysis of MADS-box transcription factor gene family in wild emmer wheat (Triticum turgidum subsp. dicoccoides)
Source: PLoS One. 2024 Mar 7;19(3):e0300159. doi: 10.1371/journal.pone.0300159 (PMC10919676; doi:10.1371/journal.pone.0300159)

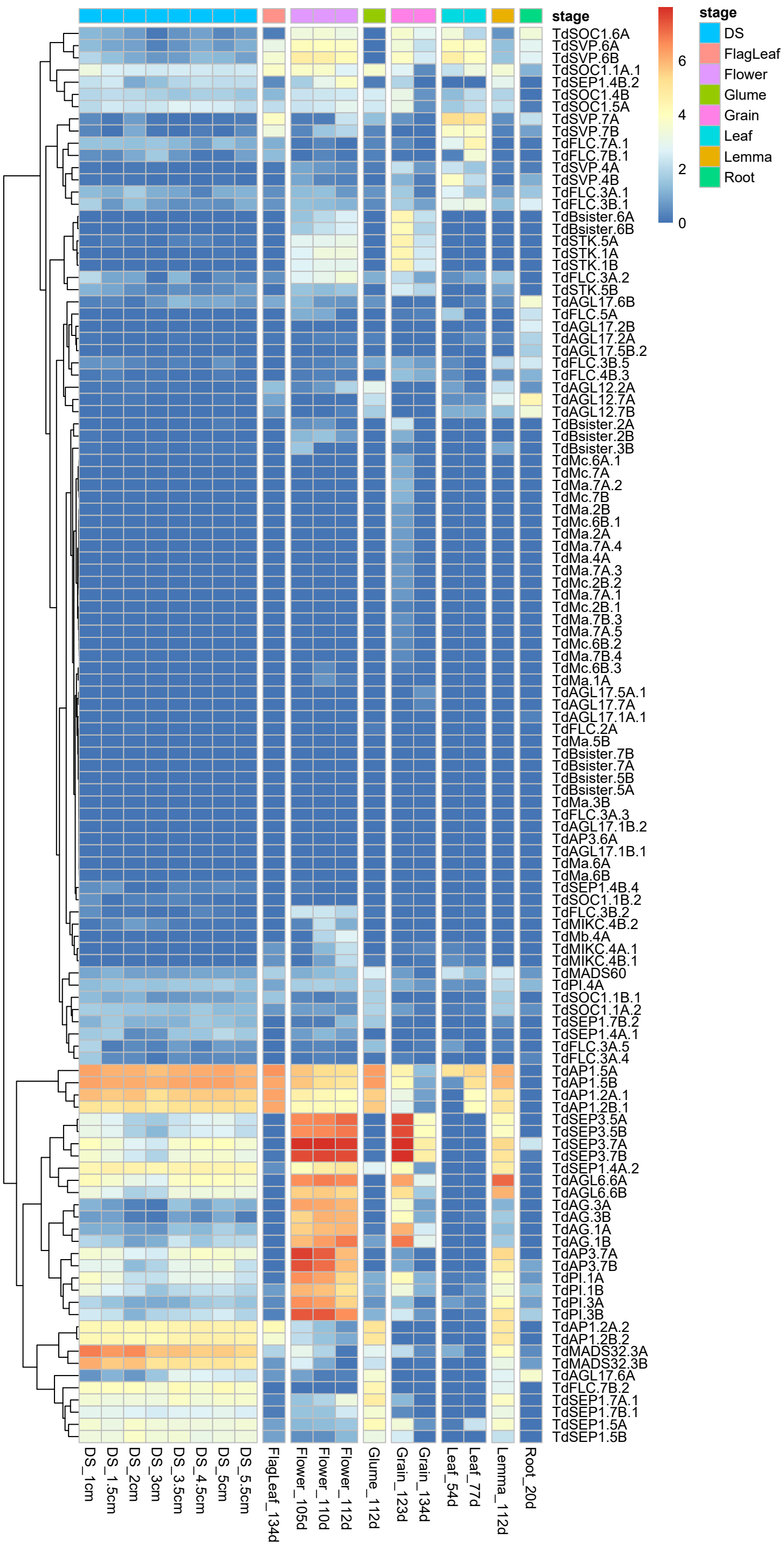

Supplement: S2 Fig — The heat map represents mean expression of 117 T. dicoccoides MADS-box genes in different tissues and developmental stages. (PDF) [file pone.0300159.s002.pdf]
